# Supplementary material for: Elicitation of Novel Trichogenic-Lipid Nanoemulsion Signaling Resistance Against Pearl Millet Downy Mildew Disease
Source: Biomolecules. 2019 Dec 23;10(1):25. doi: 10.3390/biom10010025 (PMC7022861; doi:10.3390/biom10010025)
Supplement: Supplementary file 1 [file biomolecules-10-00025-s001.pdf]

Supplementary Materials

Elicitation of novel Trichogenic-lipid nanoemulsion signaling resistance against pearl millet downy mildew disease

Boregowda Nandini[1], Hariprasad Puttaswamy[2], Harischandra Sripathy Prakash[1], Shivakanthkumar Adhikari[3], Sudisha Jogaiah[3]* and Geetha Nagaraja[1]

[1] Department of Studies in Biotechnology, University of Mysore, Manasagangotri, Mysuru 570 006, Karnataka, India; bnandini2010@gmail.com (N.B.); hasriprakash@gmail.com (H.S.P);

[2] Centre for Rural Development and Technology, Indian Institute of Technology Delhi, Hauz Khas, New Delhi 110016, India; phimprovement@rediffmail.com

[3] Laboratory of Plant Healthcare and Diagnostics, PG Department of Biotechnology and Microbiology, Karnatak University, Pavate Nagar, Dharwad 580 003, Karnataka, India; adhikariklr93@gmail.com

* Correspondence: jsudish@kud.ac.in (S.J.); geetha@appbot.uni-mysore.ac.in (G.N.); Tel.: +91-836-2779533 (S.J.); +91-0821-2419462 (G.N.); Fax: +91-836-2747884(S.J.)

Fig. 1. Specificity of qRT-PCR amplicons @ annealing temperature of 60°C.

Fig. 2. Amplification plot and Melt curve (Tm) of specific target genes.

Table 1. Details of the reference genes and target genes

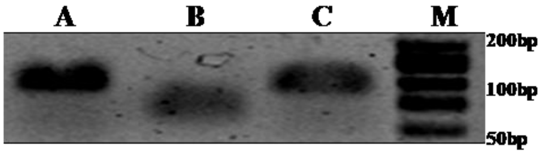

S. Fig. 1 Specificity of qRT-PCR amplicons @ annealing temperature of 60°C.

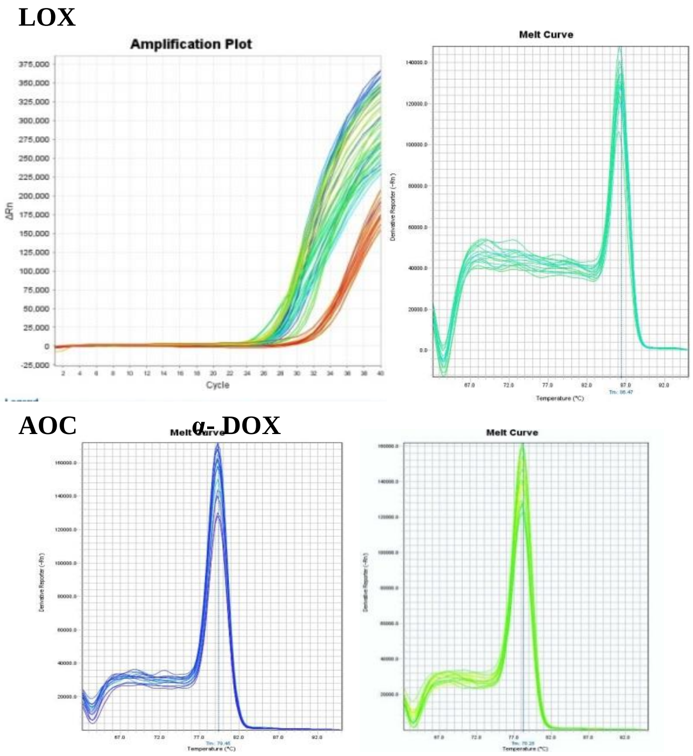

S. Fig.2. Amplification plot and Melt curve (Tm) of specific target genes.

S. Table 1. Details of the reference genes and target genes

Sl. No	Gene	Gene symbol	Primers (5′-3′)	Amplicon length(bp)
1	**Glyceraldeyde-3-phosphate dehydrogenase**	**GAPDH (reference gene)**	F - TGCCTTGCTCCCCTTGCTAA<br>R - CAGCCCTTCCACCTCTCCAG	**139**
2	**Lipoxygenase**	**LOX**	F – TTCGACTGGGAGGTGGAGAA<br>R - GGTACGTATCGTTGGAGAAG	**159**
3	**Allene oxide cyclase**	**AOC**	F – TACGGCCAGGTCAAGCTCAA<br>R - TGGTGTAGTTGTCGAGGGATGC	**184**
4	**α-Dioxygenase**	**α-DOX**	F – ATCCTGCTGCGTCCAAGTTT<br>R - ACAATCACGCCCAGAAACGA	**168**
